# ADHD and associated psychopathology in older adults in a German community sample

**DOI:** 10.1007/s00702-022-02584-4

**Published:** 2023-01-08

**Authors:** Marcus Müller, Daniel Turner, Steffen Barra, Michael Rösler, Wolfgang Retz

**Affiliations:** 1 Department of Psychiatry and Psychotherapy, University Medical Center of the Johannes Gutenberg-University, Mainz, Germany; 2 Institute for Forensic Psychology and Psychiatry, Saarland University, Homburg, Germany

**Keywords:** Attention deficit hyperactivity disorder, Psychopathology, Prevalence, Self-report, Observer report

## Abstract

Attention deficit hyperactivity disorder (ADHD) is still a neglected disorder in older adults. The aim of the present study was to examine the prevalence and symptomatology of ADHD and associated psychopathology in adults aged 40–80 years in a German community sample. We examined 539 participants in two age groups: (1) 40–59 years old (*n* = 256) and (2) 60–80 years old (*n* = 283). To assess ADHD in both childhood and adulthood as well as current psychopathological impairments, we used self-report instruments and corresponding observer reports. We examined group differences between age groups and between ADHD and non-ADHD groups. The prevalence of ADHD in the total sample was 2.6% with no significant differences between the two age groups (40–59 years: 3.1% vs. 60–80 years: 2.1%). Although differences emerged in impulsivity/emotional lability and self-concept problems, overall ADHD symptom ratings did not differ between the age groups. The ADHD group showed more psychopathological peculiarities compared to individuals without ADHD with medium-to-large effect sizes. Self-reports and observer reports showed good concordance in the assessment of ADHD and comorbid psychopathological symptoms. Regarding current ADHD symptomatology, in 92.1%, self-report was corroborated by observer's information. Our findings underline that ADHD symptoms are relevant across the lifespan. Augmenting self-reports with observer reports could increase the assessment quality of ADHD. For successful treatment, clinicians should also focus on additional psychopathological impairments and comorbidities in older adults with ADHD.

## Introduction

For a considerable period of time, it had been assumed that attention deficit hyperactivity disorder (ADHD) was just a childhood disorder; however, meanwhile, it is mostly conceptualized as a neurodevelopmental disorder with early onset that can persist into adulthood with both psychopathological symptoms and functional impairments (Faraone et al. 2021). About 15% of childhood ADHD cases show full symptomatology in adulthood, and in more than every second case, there are at least some continuing ADHD symptoms (Fayyad et al. 2007; Faraone et al. 2006). Moreover, recent longitudinal studies have found evidence for an adult-onset ADHD type; meaning individuals with adult ADHD who have never been diagnosed with ADHD during childhood (Moffitt et al. 2015; Agnew-Blais et al. 2016; Caye et al. 2016).

Prevalence estimations for childhood ADHD average around 7% (Thomas et al. 2015; Polanczyk et al. 2015). The rate of adult ADHD is lower with around 3% (Fayyad et al. 2007; Kessler et al. 2006; Simon et al. 2009; Song et al. 2021). Most previous research has not found any decline of prevalence estimates with increasing age within adulthood, and just as throughout adulthood in general, the ADHD prevalence in older adults (e.g., above 50 years of age) was estimated to be around 3% (Dobrosavljevic et al. 2020; Goodman et al. 2016; Guldberg-Kjar and Johansson 2009; Kooij et al. 2016; Michielsen et al. 2012; Sharma et al. 2021; Torgersen et al. 2016). However, a recent global systematic review and meta-analysis questioned this view, finding a decline in ADHD prevalence during adulthood irrespective of childhood or adulthood onset and a prevalence of only 0.8% in the age group 60 and above (Song et al. 2021).

Inattention, hyperactivity, and impulsivity are the three core symptoms of ADHD. However, their manifestation seems to change across the lifespan. In childhood and adolescence, ADHD is more prevalent among boys with a ratio of about 2:1, and boys show more hyperactive behaviors and more behavioral and cognitive impulsivity (especially deficits in motor inhibition and cognitive flexibility) than girls (Loyer Carbonneau et al. 2021; Dalsgaard et al. 2015). In adults, research has suggested a nearly balanced gender ratio, which accounts for adult ADHD in general and for older ADHD patients in particular (Dobrosavljevic et al. 2020; Michielsen et al. 2012; Song et al. 2021). In both men and women, hyperactivity and impulsivity decline in adulthood, whereas inattention remains nearly unchanged (Callahan & Plamondon 2019). Moreover, adult ADHD shows additional symptoms, such as emotional dysregulation, problems with self-concept, low self-esteem, low self-efficacy, or low sense of mastery (Christiansen et al. 2014; Corbisiero et al. 2013; Hirsch et al. 2018; Retz et al. 2012). Especially, untreated ADHD often leads to severe and chronic functional impairments in major life domains, e.g., education, work, relationships, friendships, and leisure activities (Able et al. 2007; Arnold et al. 2020; Barkley 2006; Fayyad et al. 2007; Halmøy et al. 2009; Holst and Thorell 2020). Furthermore, adults with ADHD show a higher tendency for antisocial behaviors and commit crimes more often than peers without ADHD, resulting in a prevalence rate of ADHD in prison samples of around 25% (Baggio et al. 2018; Retz et al. 2021; Retz and Rösler 2009; Sebastian et al. 2019; Young and Cocallis 2021).

In comparison to participants without ADHD, the psychosocial functioning of older adults with ADHD is reduced as well, but the impairments are comparable to those found in younger adults with ADHD (Philipp-Wiegmann et al. 2016; Thorell et al. 2019). For example, older adults with ADHD report about more partnership problems, higher rates of being divorced or never being married, more emotional and social loneliness, more financial problems and lower lifetime productivity, as well as a reduced health-related quality of life and reduced satisfaction with life compared to older individuals without ADHD (Michielsen et al. 2015; Brod et al. 2012; Lensing et al. 2015). Furthermore, adult ADHD does rarely occur without other comorbid psychiatric symptoms and most adults with ADHD suffer from additional psychiatric disorders during their lifetime (Simon et al. 2009; de Zwaan et al. 2012). Thereby, adult ADHD is often accompanied by mood disorders (depression, bipolar disorder), anxiety disorders, substance use disorders, autism spectrum disorder, post-traumatic stress disorder, and personality disorders (Bramhan et al. 2012; Michielsen et al. 2013; Sobanski et al. 2007; Young and Cocallis 2021). To ensure that adults with ADHD get the right treatment, clinicians need to pay extra attention on assessing additional psychopathology and comorbidities, as there is an increased risk for misinterpreting symptoms and misdiagnosing adult ADHD.

Usually, assessment of adult psychopathology relies largely on self-reports (e.g., questionnaires and interviews). Information from close persons who know the assessed person well is often only collected if self-ratings are questionable, e.g., in cases of severe psychiatric disorders like psychosis, cognitive limitations, or if the assessed person is interested in distorting the information, e.g., in forensic contexts. As information from different people displays different perspectives, assessments for clinical and research purposes benefit by augmenting self-ratings with observer ratings (Achenbach et al. 2005). Similarities in the reports could identify particularly important aspects, whereas differences could identify problems that may have been unrecognized without the consideration of multiple sources. In the case of mental illnesses in general and ADHD in particular, symptoms often only become apparent in interpersonal contact and are often not seen as problematic by the affected persons themselves. The diagnosis of mental disorders is subject to a self-report bias, especially when limited to self-report questionnaires. With regard to main symptoms of adult ADHD correlations between Conners' adult ADHD rating scale self- and observer reports, studies found correlations of *r* = 0.40–0.53 (inattention/memory), *r* = 0.52–0.70 (Hyperactivity/restlessness), *r* = 0.45–0.61 (impulsivity/emotional lability), and *r* = 0.38–0.52 (problems with self-concept) (Christiansen et al. 2014; Alexander and Liljequist 2016).

Although there are a growing number of older adults (40 years and older) diagnosed with ADHD, a knowledge gap remains in the research focusing on this population. The prevalence of ADHD in older adults and psychiatric comorbidities have been previously studied. Nevertheless, the state of research on this topic is still insufficient, especially compared to what we know in younger people with ADHD. The present study aimed to replicate previous studies and enlarge the knowledge of ADHD in older adults. We examined a large community-based sample of adults at the age of 40–80 years for ADHD symptomatology and additional psychopathological impairments. We used self-report information as well as information from close persons who knew the assessed person well. Based on the current literature, we expected a prevalence rate of adult ADHD around 3% with a slightly lower rate in the group of the 60–80 years old compared to the 40–59 years old. We further hypothesized that ADHD symptomatology would differ in the two age groups, e.g., in terms of lower impulsivity and hyperactivity in the older age group. Besides ADHD, we expected several additional psychopathological impairments, especially depressive and anxiety symptoms, and hypothesized that ADHD groups would show a higher load of psychopathological symptoms in general than non-ADHD participants. Furthermore, we expected a good concordance of self-ratings and observer ratings, but with differences in intrapsychic aspects such as self-concept problems.

## Materials and methods

### Procedure and participants

Participants for the present study were recruited from the sample of the Gutenberg Brain Study (GBS). The GBS is ongoing and started 2014. It primarily aimed at studying the genetic and environmental determinants of brain health. The GBS sample is a population-based, non-clinical sample of adults living in Mainz, the capital city of Rhineland-Palatinate, Germany. Only participants with sufficient knowledge of the German language, without learning disabilities, and without severe mental or neurological disorders were included. Within the present study, an invitation letter including the study information, a consent form to sign, and the study materials was sent out twice via mail to all participants of the GBS who agreed to be contacted for future studies. The participants were asked to fill out different paper–pencil self-report questionnaires at home. Additionally, included participants were asked to hand over the paper–pencil observer-report forms to a close relative or friend. Filled-out questionnaires were sent back to the study authors via mail. The ethics committee at the Rhineland-Palatinate state chamber of physicians approved our study protocol (No 837.128.17–10,962). Altogether, 2342 individuals from the initial GBS sample were asked to participate in the present study. Participants who sent back incomplete questionnaires were excluded. The present study included data of *N* = 539 participants between 40 and 80 years (53.1% females) in two age groups, 40 and 59 years (*n* = 256) and 60 and 80 years (*n* = 283); thus, there was a participation rate of 23.0%.

### Questionnaires

Wender Utah Rating Scale-short version (WURS-K): Childhood ADHD was retrospectively assessed by the German short version of the WURS (Ward et al. 1993), a self-rating questionnaire for the estimation of ADHD symptoms within the age of 8–10 years (Retz-Junginger et al. 2002; 2003). The WURS-K consists of 21 symptom items and 4 control items (not compatible with the construct) to be rated on a five-point Likert scale (0 = not at all to 4 = severe). The 21 symptom items are summed up to a total childhood ADHD score and a score equal or above the cut-off value of 30 indicates clinically relevant childhood ADHD symptomatology. Good psychometric properties were proven for the WURS-K, e.g., an internal consistency of Cronbach’s *α* = 0.91 (Stieglitz 2000) and a test–retest reliability of *r* = 0.90 (Rösler et al. 2008).

Conners’ Adult ADHD Rating Scales-short version (CAARS-S:S & CAARS-O:S): Current ADHD was assessed by the German version of the CAARS short form (Christiansen et al. 2014), a 26-item-rating scale for ADHD symptoms in adults aged 18 years and above. We administered both the self-rating questionnaire (CAARS-S:S) and the corresponding observer form completed by a close person, e.g., the spouse (CAARS-O:S). Symptoms are rated on a four-point Likert scale (0 = not at all/never to 3 = very much/very frequently). Besides the ADHD-Index, the CAARS short forms measure the symptoms on the four factors inattention/memory problems, hyperactivity/restlessness, impulsivity/emotional lability, and problems with self-concept. *T* values ≥ 65 indicate clinically relevant current ADHD symptomatology. The internal consistency ranged from Cronbach`s *α* = 0.80 to 0.85 for the self-rating form and from *α* = 0.83 to 0.89 for the observer’s report. Test–retest reliability ranged from *r* = 0.85 to 0.91 (Christiansen et al. 2014).

Older Adult Self-Report (OASR): The OASR is a self-report instrument to measure psychopathology in adults aged 60 years and above. The older adults rate their cognitive, emotional, behavioral, and social problems on 113 problem items. Each item is rated on a three-point Likert scale (0 = not true, 1 = somewhat or sometimes true, or 2 = very true or often true). The questionnaire provides a total problems score (a dimensional scale of general psychopathology), a personal strengths score, seven syndrome scales (anxious/depressed, worries, somatic complaints, functional impairment, memory/cognition problems, thought problems, and irritable/disinhibited), and six DSM-oriented scales (depressive problems, anxiety problems, somatic problems, dementia problems, psychotic problems, and antisocial personality problems). We considered only those scales with counterparts for the group aged 18–59 years. *T* values ≥ 60 or ≥ 65 indicate current clinically relevant symptomatology. The generalizability of OASR syndromes has been studied across 20 societies (Ivanova et al. 2020; Rescorla et al. 2022). Test–retest correlations were *r* = 0.95 for the total problems scale, *r* = 0.74 to 0.94 on the syndrome scales, and *r* = 0.78 to 0.93 for the DSM-oriented scales (Achenbach et al. 2004). The internal consistency of the total problems scale was nearly perfect with Cronbach`s *α* = 0.96, on the syndrome scales *α* ranged from 0.69 to 0.92 and on the DSM-oriented scales from 0.63 to 0.88 (Achenbach et al. 2004).

Adult Self-Report (ASR): The ASR is a 126-item self-report questionnaire assessing psychopathology in adults (18–59 years). It provides a total problems score, the personal strengths score, scores for eight syndrome scales (anxious/depressed, withdrawn, somatic complaints, thought problems, attention problems, aggressive behavior, rule-breaking behavior, and intrusive behavior), and for seven DSM-oriented scales (depressive problems, anxiety problems, somatic problems, avoidant personality problems, attention deficit/hyperactivity problems, and antisocial personality problems). To ensure comparability, we applied only those scales with counterparts for groups aged 60 years and above. The items are rated on a three-point Likert scale (0 = not true, 1 = somewhat or sometimes true, and 2 = very true or often true). *T* values ≥ 60 or ≥ 65 indicate current clinically relevant symptomatology. The ASR show good psychometric properties. For the DSM-oriented scales, the mean test–retest reliability was *r* = 0.83, and for the empirically based scales, it was *r* = 0.88 (Achenbach and Rescorla 2003). The mean internal consistency of the empirically based scales was good with Cronbach`s *α* = 0.83 and consistency score of the DSM-oriented scales was Cronbach`s *α* = 0.78 (Achenbach and Rescorla 2003).

### Statistical analyses

All statistical analyses were conducted using IBM SPSS version 26.0. Descriptive group differences between the 40–59 years old and the 60–80 years old were investigated using *χ*^2^ analyses for categorical variables. Missing values were excluded from the calculation. Cramér’s *V* effect size values of 0.10, 0.30, and 0.50 represented small, medium, and large effects, respectively (Cohen 1988). The level of significance was adjusted for accumulation of type-I error using the False Discovery Rate (FDR) (Benjamini and Hochberg 1995). Thereby, the FDR is less conservative than the traditionally used Bonferroni correction; however, it was suggested to prefer the FDR over the Bonferroni method particularly in health and medical research (Glickman et al. 2014). To compare self-reports and observer’s reports, we conducted product–moment correlations. Pearson *r* correlations with values of 0.10, 0.30, and 0.50 represented small, medium, and large effects (Cohen 1988). In addition, we examined the associations between ADHD symptomatology and psychopathological impairments (e.g., general psychopathology, depressive problems, and anxiety problems) as well as personal strengths using Pearson’s correlations. We also investigated descriptive group differences building categorical variables for (1) clinically relevant ADHD symptoms in childhood (WURS-K score ≥ 30), and (2) clinically relevant ADHD symptoms in adulthood (CAARS-S:S ADHD-Index T value ≥ 65). Cut-off values were determined based on respective test manuals. For linear variables (ASR/OASR t values), we conducted *t* tests with Cohen’s *d* as effect size (0.20 = small, 0.50 = medium, and 0.80 = large).

## Results

The mean age of the total sample (*N* = 539, 53.1% females) was 59.63 years (*SD* = 9.15, range 40–80). Regarding female–male ratio, the two age groups did not differ (*χ*^2^(1) = 1.40, *p* = 0.24, *V* = 0.05). No significant differences were found between the two age groups for relationship status (*χ*^2^(1) = 1.48, *p* = 0.22, *V* = 0.05). Approximately four out of five older adults lived in a solid partnership (40–49 years: 83.2% vs. 60–80 years: 79.2%). However, the younger age group showed higher educational levels (*χ*^2^(1) = 5.42, *p* = 0.02, *V* = 0.10). In the age group of 40–59 years old (*n* = 256), 14.5% of the participants showed low education (≤ 10 school years) versus 22.3% in the age group of 60–80 years old (*n* = 283). Descriptive statistics of the study sample are presented in Table 1.

**Table 1:** Sample characteristics (*N* = 539)

	Total (*N* = 539)	40–59 years (*n* = 256)	60–80 years (*n* = 283)	*χ*^2^ (1)	*p*	Cramér’s *V*
Age *M (SD)*	59.63 (9.15)	51.80 (5.55)	66.70 (5.10)			
Gender						
Female	286 (53.1%)	129 (50.4%)	157 (55.5%)	1.40	0.24	0.05
Male	253 (46.9%)	127 (49.6%)	126 (44.5%)			
Highest education						
≤ 10 school years	100 (18.6%)	37 (14.5%)	63 (22.3%)	5.42	0.02	0.10
> 10 school years	439 (81.4%)	219 (85.5%)	220 (77.7%)			
Relationship status						
Solid partnership	437 (81.1%)	213 (83.2%)	224 (79.2%)	1.48	0.22	0.05
Single	100 (18.6%)	42 (16.4%)	58 (20.5%)			
n.a	2 (0.4%)	1 (0.4%)	1 (0.4%)			

*V* ≥ 0.10 = small, *V* ≥ 0.30 = medium, *V* ≥ 0.50 = large.

### Prevalence of ADHD symptomatology in the two age groups

In the total sample (*N* = 539), the prevalence of self-reported clinically relevant ADHD symptoms in childhood (WURS-K score ≥ 30) was 8.9% (95% CI 6.5%–11.5%). The prevalence of self-reported clinically relevant ADHD symptoms in adulthood (CAARS-S:S ADHD-Index *t* value ≥ 65) was 7.4% (95% CI: 5.2%–9.8%). Considerably less individuals reported about ADHD symptomatology in both childhood and adulthood (2.6%, 95% CI 1.3%–3.9%). Neither the ADHD prevalence in childhood nor the prevalence in adulthood significantly differed between the two age groups (see Table 2).

**Table 2:** Prevalence of self-reported and observer-reported ADHD in childhood and adulthood and comparison of the two age groups (*N* = 539)

	Total (*N* = 539)		40–59 years (*n* = 256)		60–80 years (*n* = 283)		*χ*^2^ (1)	*p*^*2*^	Cramér’s *V*
	*n*	% (95% CI)	*n*	% (95% CI)	*n*	% (95% CI)			
ADHD in childhood (WURS-K ≥ 30)	48	8.9 (6.5–11.5)	28	10.9 (7.7–14.8)	20	7.1 (4.2–10.2)	2.48	0.12	0.07
ADHD in adulthood (t scores ≥ 65)									
CAARS-K S (self-rating)									
Inattention/memory problems	36	6.7 (4.6–9.1)	20	7.8 (4.7–10.9)	16	5.7 (3.2–8.5)	1.01	0.32	0.04
Hyperactivity/restlessness	40	7.4 (5.4–9.8)	24	9.4 (5.9–13.3)	16	5.7 (3.2–8.5)	2.71	0.10	0.07
Impulsivity/emotional lability	37	6.9 (4.8–9.1)	23	9.0 (5.5–12.9)	14	4.9 (2.5–7.8)	3.43	0.06	0.08
Problems with self-concept	57	10.6 (8.2–13.4)	34	13.3 (9.4–17.6)	23	8.1 (5.3–11.3)	3.78	0.05	0.08
ADHD-Index	40	7.4 (5.2–9.8)	23	9.0 (5.9–12.5)	17	6.0 (3.2–8.8)	1.73	0.19	0.06
ADHD in Child & Adulthood (self-rating)									
WURS-K & inattention/memory problems	10	1.9 (0.7–3.2)	6	2.3 (0.8–4.3)	4	1.4 (0.4–2.8)	0.64	0.42	0.03
WURS-K & hyperactivity/restlessness	14	2.6 (1.5–3.9)	10	3.9 (1.6–6.3)	4	1.4 (0.4–2.8)	3.30	0.07	0.08
WURS-K & impulsivity/emotional lability	12	2.2 (1.1–3.5)	9	3.5 (1.6–5.9)	3	1.1 (0.0–2.5)	3.72	0.05	0.08
WURS-K & problems with self-concept	14	2.6 (1.3–4.1)	9	3.5 (1.6–5.9)	5	1.8 (0.4–3.2)	1.63	0.20	0.06
WURS-K & ADHD-Index	14	2.6 (1.3–3.9)	8	3.1 (1.2–5.5)	6	2.1 (0.7–3.9)	0.54	0.46	0.03
ADHD in adulthood (t scores ≥ 65)									
CAARS-K O (rating by others)^1^									
Inattention/memory problems	15	3.2 (1.7–4.9)	12	5.0 (2.5–7.9)	3	1.3 (0.0–3.1)	4.89	0.03	0.10
Hyperactivity/restlessness	27	5.8 (3.9–7.9)	18	7.4 (4.5–11.2)	9	4.0 (1.8–6.7)	2.49	0.11	0.07
Impulsivity/emotional lability	12	2.6 (1.3–4.1)	8	3.3 (1.2–5.8)	4	1.8 (0.4–3.6)	1.07	0.30	0.05
Problems with self-concept	28	6.0 (4.1–8.2)	21	8.7 (5.4–12.4)	7	3.1 (0.9–5.8)	6.35	0.01	0.12
ADHD-Index	19	4.1 (2.4–6.0)	13	5.4 (2.5–8.3)	6	2.7 (0.9–4.9)	2.16	0.14	0.07
ADHD in Child & Adulthood (rating by others)^1^									
WURS-K & inattention/memory problems	2	0.4 (0.0–1.1)	1	0.4 (0.0–1.2)	1	0.4 (0.0–1.3)	0.00	0.96	0.00
WURS-K & hyperactivity/restlessness	4	0.9 (0.2–1.7)	4	1.7 (0.4–3.3)	0	0.0 (0.0–0.0)	3.74	0.05	0.09
WURS-K & impulsivity/emotional lability	0	0.0 (0.0–0.0)	0	0.0 (0.0–0.0)	0	0.0 (0.0–0.0)			
WURS-K & problems with self-concept	0	0.0 (0.0–0.0)	0	0.0 (0.0–0.0)	0	0.0 (0.0–0.0)			
WURS-K & ADHD-Index	2	0.4 (0.0–1.1)	2	0.8 (0.0–2.1)	0	0.0 (0.0–0.0)	1.86	0.17	0.06

*V* ≥ 0.10 = small, *V* ≥ 0.30 = medium, *V* ≥ 0.50 = large.

^1^*n* = 466

^2^After adjusting the *p* values for multiple testing with the FDR method, none of the differences between the two age groups were significant

Adults from the younger age group showed significantly more problems with self-concept than participants from the older age group, while no differences were found in the other CAARS-S:S subscales (see Table 2). Finally, the observer’s reports (CAARS-O:S) suggested somewhat lower rates of adult ADHD in both age groups [40–59 years: 5.4% (95% CI 2.5–8.3%); 60–80 years: 2.7% (95% CI 0.9–4.9%)], but again no significant differences. However, in the younger age group, significantly more problems with inattention/memory and with self-concept were reported (see Table 2). After adjusting the *p *values for multiple testing with the FDR method, none of the differences between the two age groups were significant.

With regard to gender differences, 7.0% of the examined females (*n* = 286) and 11.1% of the males (*n* = 253) reported clinically relevant ADHD symptomatology in childhood. No statistical significant gender effect was found (*χ*^2^(1) = 2.78, *p* = 0.10, *V* = 0.07). In adulthood, 7.7% of the women and 7.1% of the men reported clinically relevant ADHD symptoms, also with no significant difference (*χ*^2^(1) = 0.07, *p* = 0.80, *V* = 0.01). Both ADHD symptomatology in childhood and adulthood have been found in 2.4% of the examined women and 2.8% of the men; again, differences were not statistically significant (*χ*^2^(1) = 0.05, *p* = 0.82, *V* = 0.01). However, when comparing the two age groups, a significant gender difference was found in the group of the 40–59 year old adults (*χ*^2^(1) = 5.99, *p* = 0.01, *V* = 0.15). In this younger age group, 6.2% of the females but 15.7% of males reported childhood ADHD symptoms. In the age group of the 60–80 years old (male: 6.3% vs. female: 7.6%), no significant difference was found (*χ*^2^(1) = 0.18, *p* = 0.67, *V* = 0.03). With regard to adult ADHD, 6.2% females versus 11.8% males of the 40–59 year old adults reported current clinical relevant ADHD symptoms, with no significant gender difference (*χ*^2^(1) = 2.46, *p* = 0.12, *V* = 0.10). In the older age group (60–80 years) the proportion of females with self-rated current ADHD symptoms (8.9%) differed significantly from the proportion of males (3%) (*χ*^2^(1) = 5.29, *p* = 0.02, *V* = 0.14). In the group of the 40–59 years old adults, both clinical relevant ADHD symptomatology in childhood and in adulthood were found significantly more often in males than in females (5.5% vs. 0.8%) (*χ*^2^(1) = 4.74, *p* = 0.03, *V* = 0.14). In the group of the 60–80 year old adults, the gender ratio also differed significantly (*χ*^2^(1) = 4.92, *p* = 0.03, *V* = 0.13) and the proportion of women who met the criteria (3.8%) was higher than the proportion of men (0.0%).

### Correlations between self- and observer reports

Regarding current ADHD symptomatology, the correlations between self-reports and observer’s reports showed medium-to-large effects. In 92.1% of the 466 cases in which both self- and observer ratings were present, observers and participants were unanimous in their assessment of whether a current clinical relevant ADHD symptomatology exists. In the total sample, *r* coefficients were between 0.39 and 0.44. In the group of the 40–59 year old adults, *r* coefficients were between 0.40 and 0.53 and in the group of the 60–80 year old adults between 0.35 and 0.42 (see Table 3).

**Table 3:** Correlations between self- and observer reports concerning ADHD symptomatology

	Total				40–59 years				60—80 years			
	*M (SD)*				*M (SD)*				*M (SD)*			
	Self	Observer	*r*	*p*	Self	Observer	*r*	*p*	Self	Observer	*r*	*p*
	(*n* = 539)	(*n* = 466)			(*n* = 256)	(*n* = 242)			(*n* = 283)	(*n* = 224)		
Inattention/memory problems	47.75 (10.06)	46.39 (7.82)	0.44	< 0.001	47.42 (10.37)	46.75 (8.41)	0.47	< 0.001	48.05 (9.78)	46.01 (7.12)	0.42	< 0.001
Hyperactivity/restlessness	47.73 (10.31)	47.24 (8.98)	0.42	< 0.001	48.41 (10.59)	47.79 (9.29)	0.47	< 0.001	47.12 (10.03)	46.65 (8.61)	0.35	< 0.001
Impulsivity/emotional lability	47.92 (8.57)	46.42 (7.51)	0.39	< 0.001	48.27 (9.40)	47.13 (8.31)	0.40	< 0.001	47.60 (7.74)	45.67 (6.48)	0.35	< 0.001
Problems with self-concept	49.02 (12.41)	46.64 (9.91)	0.49	< 0.001	50.21 (13.51)	47.67 (11.06)	0.53	< 0.001	47.95 (11.25)	45.54 (8.38)	0.40	< 0.001
ADHD-Index	47.86 (10.33)	46.17 (8.37)	0.39	< 0.001	48.32 (11.20)	46.92 (9.01)	0.41	< 0.001	47.45 (9.48)	45.36 (7.54)	0.35	< 0.001

*r* = 0.10 = small, *r* = 0.30 = medium, and *r* = 0.50 = large.

### Differences between ADHD and non-ADHD participants concerning comorbid psychopathological symptoms

We found a statistically significant difference between the ASR/OASR total problems score (general psychopathology) and on all included subscales between the childhood ADHD group (*n* = 48) and the non-ADHD childhood group (*n* = 491) (see Table 4). Participants who reported about an ADHD symptomatology in childhood had significantly more psychopathological symptoms during adulthood than those participants who did not report about ADHD symptomatology in childhood. Effect sizes were medium to large.

**Table 4:** Differences between ADHD (in childhood and adulthood) versus Non-ADHD and psychopathological impairments (*N* = 539)

ASR/OASR scales	ADHD child^1^		Non-ADHD child		*t*	*p*	Cohen's *d*	ADHD adult^2^		Non-ADHD adult		*t*	*p*	Cohen's *d*
	(*n* = 48)		(*n* = 491)					(*n* = 40)		(*n* = 499)				
	*M*	*SD*	*M*	*SD*				*M*	*SD*	*M*	*SD*			
Total problems	56.60	8.81	46.03	9.40	− 7.48	< 0.001	− 1.13	63.86	8.03	45.62	8.62	− 12.93	< 0.001	− 2.13
Personal strengths	49.02	7.97	52.46	7.13	3.16	0.002	0.48	47.45	8.76	52.53	7.01	4.33	< 0.001	0.71
*Syndrome scales*														
Anxious/depressed	57.56	7.29	52.60	5.16	− 6.10	< 0.001	− 0.92	64.23	9.15	52.15	4.00	− 16.08	< 0.001	− 2.64
Somatic complaints	55.46	7.46	52.44	4.88	− 3.87	< 0.001	− 0.59	60.65	10.15	52.07	3.99	− 11.06	< 0.001	− 1.82
Thought problems	57.48	7.33	53.45	5.44	− 4.73	< 0.001	− 0.72	62.38	8.62	53.12	4.85	− 10.80	< 0.001	− 1.78
*DSM-oriented scales*														
Depressive problems	57.58	8.91	52.88	5.21	− 5.52	< 0.001	− 0.84	64.50	10.44	52.40	4.09	− 15.21	< 0.001	− 2.50
Anxiety problems	55.31	6.35	52.34	4.66	− 4.07	< 0.001	− 0.62	61.75	7.19	51.87	3.83	− 14.44	< 0.001	− 2.37
Somatic problems	55.67	6.82	52.50	4.98	− 4.05	< 0.001	− 0.61	60.35	9.67	52.18	4.17	− 10.39	< 0.001	− 1.71
Antisocial personality	57.88	6.57	53.12	4.12	− 7.16	< 0.001	− 1.08	58.88	6.48	53.12	4.12	− 8.07	< 0.001	− 1.33

^1^WURS-K score ≥ 30.

^2^CAARS-S:S ADHD-Index T values ≥ 65.

*df* = 537 for all analyses.

*d* = 0.20 = small, *d* = 0.50 = medium, and *d* = 0.80 = large.

The same accounted for the ADHD adult group. Adults with ADHD symptomatology reported about significantly more psychopathological symptoms on all included subscales compared to adults without ADHD symptomatology during adulthood. Effect sizes were again medium to large (see Table 4).

## Discussion

The present study contributes to previous research of ADHD in older adults. With the use of self- and observer reports, we examined the prevalence and symptomatology of ADHD and general psychopathology in a sample of 539 adults between 40 and 80 years in a German community sample.

Previous meta-analyses have found prevalence rates for childhood ADHD around 7% (Thomas et al. 2015; Polanczyk et al. 2015). We found a prevalence rate of clinically relevant ADHD symptoms in childhood of 8.9%, which is slightly higher but still comparable to previously found numbers. As we used self-report questionnaires for the assessment of ADHD, this could explain the slightly higher prevalence. However, the concordance between the prevalence estimates found in the present study with previous studies speaks for the validity of our findings. We found a decline in the rate of ADHD from childhood to adulthood in our sample, which is also in line with previous research. Current and symptomatic adult ADHD was reported by 7.4%. Both, clinically relevant symptoms in childhood as well as in adulthood were reported by 2.6% of our sample, a rate which goes well with the current empirical knowledge about the course of ADHD along the lifespan and confirms our hypothesis of a continuous decrease of ADHD symptomatology with increasing age (Wootton et al. 2022). However, looking specifically at the group of the 60–80 years old, the rate we found (2.1%) is 2.5 times higher than the 0.8% found in the meta-analysis of 40 studies by Song et al. (2021) in this age group. This difference could be due to differing assessment methods. However, in line with the meta-analysis performed by Song et al., we also found a decrease in the ADHD prevalence with increasing age, but differences were not statistically significant. Thus, our results provide further support for the suggestion that there is a decrease in the ADHD prevalence with age, yet ADHD persists in a significant number of older people.

Regarding the current main symptoms of ADHD, the 40–59 years old and the 60–80 years old showed a very similar core symptomatology. However, our results indicated a small trend toward a lower symptom burden in the older age group. The only exception were problems with self-concept. In the younger age group, both via self-report and from the observer perspective, there was a higher rate of self-concept problems than in the older age group, which was operationalized as difficulties that may include bad social relationships, lower self-esteem, and lower self-confidence. However, it must be noted that differences between the two age groups in terms of self-concept problems were only found when it was not taken into account whether clinically relevant ADHD symptoms were already present in childhood. Furthermore, the differences disappeared after adjusting the *p* values for multiple testing. Reasons for differences regarding self-esteem and self-confidence between ADHD age groups could be changes in the living conditions in this period of life, for example, that more people were already retired and no longer working in the older age group. It seems plausible that the restricted work performance due to ADHD has a less negative effect on people suffering from ADHD in this phase of life and that other everyday aspects such as health care and age-related physical impairments come to the fore. It should also be taken into account that with increasing age, the strategies for dealing with ADHD-related impairments could be optimized.

In the group of individuals with ADHD symptoms in childhood and in adulthood, we found a lower rate of impulsivity/emotional lability in the older age group compared to the middle-aged group. Thus, more participants reported clinically relevant difficulties including impulsive acts, low frustration tolerance, quick and frequent mood changes, and feeling easily angered and irritated by people in the younger age group. Moreover, from the observer’s perspective, the two age groups differed also in hyperactivity, with the younger age group showing more difficulties like restlessness and being more fidgety than the 60–80 year old group. The decline of impulsivity and hyperactivity in ADHD patients across the lifespan is well documented by follow-up studies that observed the persistence of ADHD from childhood into adulthood (Biederman et al. 2000; Callahan and Plamondon 2019) and confirms our hypothesis of an ongoing decrease of ADHD symptoms over the lifespan. With regard to the changes in ADHD core symptoms, however, it must be pointed out that we only found small age effects and after adjusting the *p* values for multiple testing the significant differences disappeared.

Additionally to the self-reports, we were able to include a high proportion of observer reports (86%), i.e., assessments of the participant’s ADHD symptomatology by a close person like their partner. According to our hypothesis, the comparison of 466 self-report/observer report dyads showed good concordance with medium-to-large correlations. This finding fits well to correlation coefficients found in the validation study of the German version of the CAARS (Christiansen et al. 2014) which were also medium to large. Regarding every measured core symptom scale, we found somewhat higher correlations in the younger age group than in the older age group. However, there were only minor differences in the *r* coefficients. Contrary to our hypothesis, the concordance between self and observer ratings of self-concept problems was also medium to large, although these intrapsychic symptoms are harder to notice from the observer’s perspective than the other main ADHD symptoms. Furthermore, self-concept problems of adults with ADHD often stay hidden for others and the affected persons appear self-confident, although they may not be. The results of good concordance of self- and observer ratings support the multimodal approach in diagnosing psychiatric disorders. Clinicians should obtain information from several sources and different perspectives. Observer reports, especially from close persons, can provide valuable additional information. They can also be a good alternative in situations where the concerned person is unable or unwilling to provide information.

Consistent with other research findings (Loyer Carbonneau et al. 2021; Dalsgaard et al. 2015), our results showed a slight trend indicating that more men reporting clinically relevant ADHD symptoms in childhood than women. In adulthood, the ratio adjusted and there was no longer a clear gender effect in both age groups, which also fits well with the previous research findings (Dobrosavljevic et al. 2020; Song et al. 2021). It could be possible that the ADHD core symptom hyperactivity, which is more pronounced in boys than in girls, weakens with increasing age or changes in the way it is expressed. The increased urge to move is less openly acted out by adults compared to children and adults often engage in more pronounced and successful coping mechanisms regarding their hyperactivity. This change in ADHD symptomatology across the lifespan may lead to a convergence of the prevalence and a nearly balanced gender ratio in adults.

The participants with self-reported ADHD symptoms in childhood or adulthood showed a considerable higher burden of psychopathological impairments than participants without ADHD symptoms. In the childhood ADHD group, the general psychopathology score was one standard deviation higher than in the non-ADHD group. In the adult ADHD group, the difference was even nearly two standard deviations. Participants who met the criteria for clinically relevant childhood or adult ADHD symptoms additionally reported significantly more current depressive, anxiety, psychosomatic symptoms, and more thought problems. At the same time, adults with ADHD symptomatology reported fewer personal strengths, what means less resources and protective factors against the development or the maintenance of comorbidities. However, it must be noted that in the case of childhood ADHD, all scores for psychopathological impairments were still below the cut-off values for clinically relevant symptomatology. In contrast, in case of current and symptomatic ADHD, the scores were higher and in the borderline range between absence and presence of clinical relevance. Furthermore, the score for self-reported antisocial behavior problems was higher in both ADHD groups in comparison with the non-ADHD group, which confirms the scientific knowledge of a higher tendency for social inadequate behavior in adults with ADHD in comparison with persons without ADHD (Young and Cocallis 2021; Retz et al. 2021; Sebastian et al. 2019).

Eventually, our results show that ADHD is a neurodevelopmental disorder that can negatively influence an individual’s further development with unfavorable impact in nearly all areas of life. This emphasizes the great importance of accurate diagnosis and adequate treatment to improve quality of life and prevent from the development of comorbid disorders. Furthermore, if ADHD is suspected, clinicians should not only consider the typical ADHD symptoms, but also consider other psychopathological impairments.

Some limitations and several strengths must be considered in the interpretation of our results. Current diagnostic criteria of ADHD require evidence of symptom onset in childhood (prior to age 12). There is considerable concern about the ability of adults to accurately recall childhood conditions (Fayyad et al. 2007; Kessler et al. 2006) and there is a risk to under- or overestimate symptoms. There could be a self-report bias, because the symptoms are so far in the past, especially for older adults. Moreover, the diagnosis ADHD was only introduced with the DSM-III in 1980, which means that the diagnosis did not yet exist in the childhood of many participants. However, especially in the elderly, there is no feasible alternative to self-reports and ancillary sources are often unavailable. We used the WURS-K as an adequate and practical instrument for the retrospective estimation of ADHD symptoms in childhood with an 85–93% sensitivity and a 76–92% specificity for childhood ADHD (Retz-Junginger et al. 2003). To assess current and clinically relevant ADHD, we used self-reports and additional observer reports, however, including clinician-administered diagnoses would have increased the quality of the assessment of ADHD symptoms and comorbidities, which was, however, not possible in the present study.

Moreover, we compared two adult age groups. A prospective longitudinal design from childhood to old age would have allowed statements about the development of ADHD across the lifespan and into old age. We collected data from a community-based sample in Germany, which was quite large, but still too small to be a representative. It is not possible to generalize the outcomes into other societies without limitations. We recruited our sample from subjects who had given their interest in studying brain health. This may influence the results. Of these subjects, only those who also had an interest in studying mental health completed our questionnaires, which may have introduced further bias. Considering this, it is more likely that psychopathological abnormalities were overestimated in our sample. The study was not administered specifically to participants with clinical adult ADHD, so the ADHD group is relatively small and so the interpretation of the comparison of the ADHD versus the non-ADHD group is limited. For this reason, we have refrained several analyses. Future studies should investigate the difference in psychopathological distress between those patients who received treatment in childhood compared to those who did not. Because not all individuals with adult ADHD present additional psychopathological impairments in adulthood, it should also be examined which risk factors increase the likelihood of comorbid psychiatric disorders.

In conclusion, the current findings provide further evidence of the prevalence rate of adult ADHD of around 3%, even in the group of adults aged 40 to 80 years. Between elderly and middle-aged adults, there are more similarities than differences in the symptomatology of ADHD with no significant gender effects. ADHD is often accompanied by additional psychopathological symptoms and life impairments. To offer sufficient and effective treatment to older adults with ADHD, it is important to assess symptoms well and thoroughly. Augmenting observer ratings to self-ratings can make a valuable contribution. Further well-designed prospective studies in older adults with ADHD need to be conducted to broaden the current knowledge in this area.

## Data Availability

The dataset generated and analysed during the current study are available from the corresponding author on reasonable request.
